# Motor-related signals support localization invariance for stable visual perception

**DOI:** 10.1371/journal.pcbi.1009928

**Published:** 2022-03-14

**Authors:** Andrea Benucci

**Affiliations:** 1 RIKEN Center for Brain Science, Wako-shi, Japan; 2 University of Tokyo, Graduate School of Information Science and Technology, Department of Mathematical Informatics, Tokyo, Japan; Chinese Academy of Sciences, CHINA

## Abstract

Our ability to perceive a stable visual world in the presence of continuous movements of the body, head, and eyes has puzzled researchers in the neuroscience field for a long time. We reformulated this problem in the context of hierarchical convolutional neural networks (CNNs)—whose architectures have been inspired by the hierarchical signal processing of the mammalian visual system—and examined perceptual stability as an optimization process that identifies image-defining features for accurate image classification in the presence of movements. Movement signals, multiplexed with visual inputs along overlapping convolutional layers, aided classification invariance of shifted images by making the classification faster to learn and more robust relative to input noise. Classification invariance was reflected in activity manifolds associated with image categories emerging in late CNN layers and with network units acquiring movement-associated activity modulations as observed experimentally during saccadic eye movements. Our findings provide a computational framework that unifies a multitude of biological observations on perceptual stability under optimality principles for image classification in artificial neural networks.

## Author summary

Stable visual perception during eye and body movements suggests neural algorithms that convert location information—"where” type of signals—across multiple frames of reference, for instance, from retinocentric to craniocentric coordinates. Accordingly, numerous theoretical studies have proposed biologically plausible computational processes to achieve such transformations. However, how coordinate transformations can then be used by the hierarchy of cortical visual areas to produce stable perception remains largely unknown. Here, we explore the hypothesis that perception equates to the activity states of networks trained to classify “features” (e.g., objects, salient components) in the visual scene, and perceptual stability equates to robust classification of these features relative to self-generated movements, that is, a “what” type of information processing. We demonstrate in CNNs that neural signals related to eye and body movements support accurate image classification by making “where” type of computations—localization invariances—faster to learn and more robust relative to input perturbations. Therefore, by equating perception to the activity states of classifier networks, we provide a simple unifying mechanistic framework to explain the role movement signals in support of stable perception in dynamic interactions with the environment.

## Introduction

When reading this paper while sitting still at your desk, unperceived head and body adjustments, along with continuous eye movements—fixational eye movements [1]—jitter the visual image across arrays of photoreceptors in the retinas of the eyes. However, the visual scene is perceived as *still*. Similarly, when making saccadic eye movements, the visual world does not transiently become blurry; rather, our experience is characterized by a striking perceptual continuity [2]. Notably, when eye movements are counteracted, e.g., by stabilizing an image relative to retinal displacement, perception fades away [3], underscoring the necessity of movements in visual perception. Over the decades, research on perceptual stability has revealed a multiplicity of computational and physiological phenomena that operate across multiple spatial-temporal scales and brain regions (reviewed, e.g., in [4–6]).

A branch of modeling works has linked the ability to accurately recognize objects during movements—which could support perceptual stability—to invariances for translations, rotations, and expansions learned directly from the statistics of the visual inputs. This class of models, e.g., unsupervised temporal learning models [7,8] and slow feature analysis models [9–14] has found supporting evidence in psychophysical [15,16] and physiological studies [7,17,18] and has inspired deep learning approaches for unsupervised rules to learn coherent visual representations in the presence of moving stimuli e.g., contrastive embedding [19,20]. However, these models are agnostic with respect to whether retinal activations are due to objects moving in the environment or to movements of the organism, with the latter characteristically defining the phenomenon of perceptual stability. Therefore, another branch of works has hypothesized that extra-retinal signals produced during body movements, *corollary discharges* [21–24], could be used by brain networks for perceptual stabilization specifically when retinal activations are due to the movements of the eyes, head, and body, without affecting the percept of movements during changes in the environment.

Lacking a quantitative definition of what visual perception is, it has been challenging to gain a mechanistic understanding of how corollary discharges could implement perceptual stability. A body of modeling studies has cleverly circumvented this problem by reasoning that, aside from a quantitative definition, perceptual stability should implicate a transformation between spatial reference frames, correcting localization errors and spatial distortions associated to peri-saccadic visual processing, proposing approaches that differ by how extra-retinal information is combined with visual signals. Except for object reference theory, which does not include extra-retinal signals in its formalism, relying instead on the concept of “trans-saccadic memory” [25–28], other models implemented visuomotor integrations for spatial transformations according to a broad diversity of algorithms. For instance, Bayesian models for optimal (trans-saccadic) integration [29,30], remapping models [31,32], spatial re-entry models [33–35], inference models based on retinal-to-craniocentric coordinate transformations [36–41], and gain-field models [42,43]. The latter have been proposed in different flavors: in reference to multisensory integration [37,44,45], using gain fields as basis functions for the encoding of the perceptual space [45–47], or without explicit reference frame transformation, using eye position or velocity in retinocentric representation to achieve a desired motor error [39,48,49]. Collectively, gain-field and other models have been successfully used to account for a diversity of saccadic-related phenomena such as saccadic suppression of stimulus displacement [50], post-saccadic blanking [51], suppression of saccadic displacement [52], receptive-field remapping [32,53–57], establishing tight links between eye movements, remapping, and spatial attention [33–35]. However, without a quantitative definition of what visual perception is, it remains unclear how these visual-localization computations can be mechanistically used by cortical network to implement stable perception.

Here we used CNNs, intended as models of the hierarchically organized visual system [58,59], and adopted an operational definition of visual perception as activity states of networks trained to classify features in the visual scene (e.g., objects or salient parts of objects) while integrating corollary discharge signals; the latter point reflecting the necessity of self-generated movements and sensorimotor integration for perception. Perceptual stability can then be examined as an optimization problem that aims to maximize classification accuracy in the presence of self-generated movements.

We demonstrated these concepts first in the context of fixational eye movements, using CNNs trained to report feature locations of a single object (a luminance bar); we showed that when the magnitude of eye movements is commensurate to location differences that need to be discriminated, motor-related signals can resolve a critical degeneracy problem “posed” by retinotopy. Further, we examined this degeneracy-solving principle in the context of larger eye movements (saccades) and in the classification of natural images; we showed that CNNs can use corollary discharge signals to accelerate the learning of classification invariance relative to image translations reflecting self-generated movements, and to increase the robustness of learned classification relative to input noise. We concluded by examining the representational geometry defined by visual responses across layers in trained CNNs, thereby revealing that CNNs can build an invariance to self-generated saccadic shifts by forming categorical representations of natural images across layers that are aided by corollary discharge signals, and with visuomotor integration linked to neural activations bearing close resemblance to biological response modulations observed during saccadic eye movements.

## Results

### 1. Fixational eye movements

**Fig 1:**
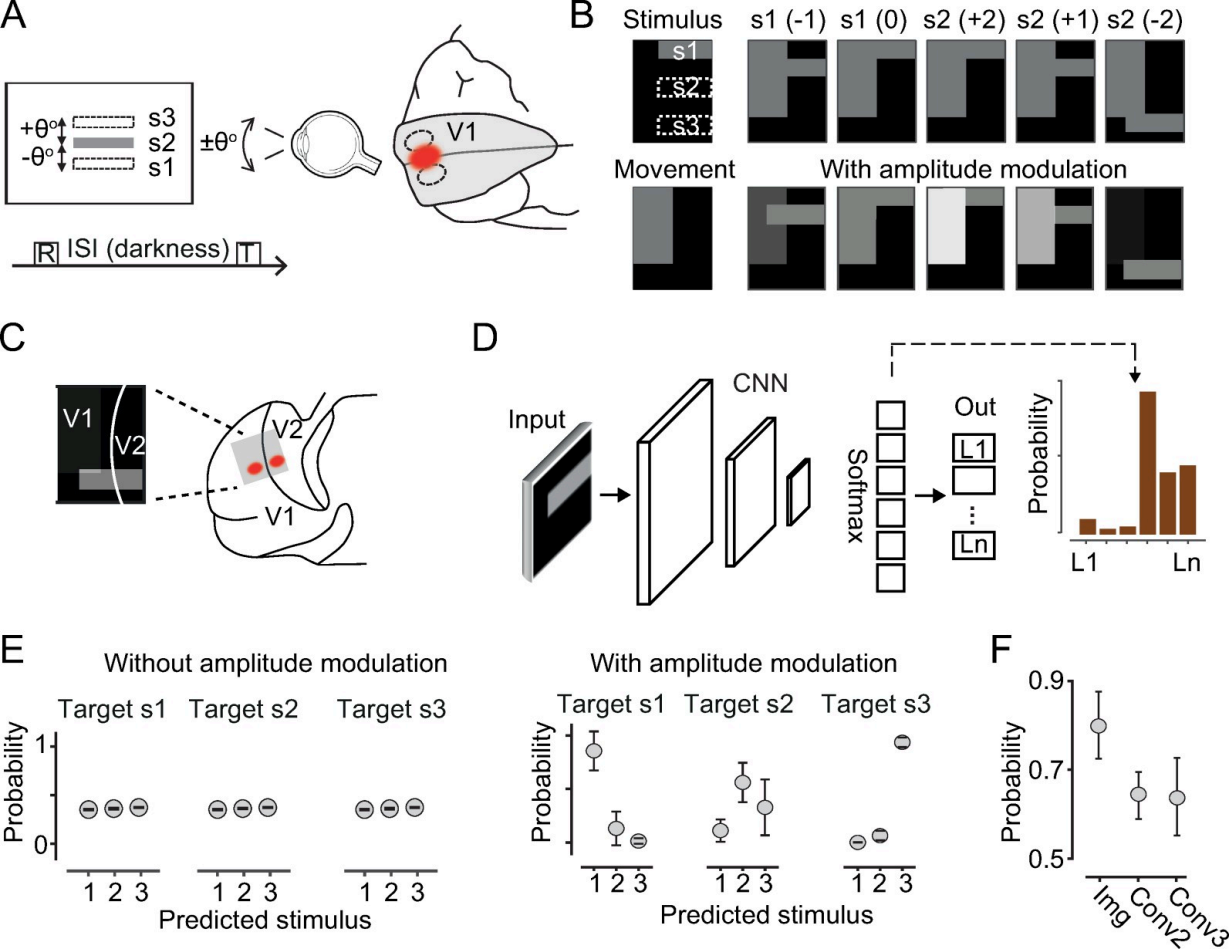
Corollary discharge signals enable localization invariance relative to image displacements. (A) Simulated psychophysical task. A participant is briefly shown a reference luminance bar (boxed “R” letter, and “S2” middle gray bar) and, after a delay in darkness (ISI), a test stimulus (T) is briefly presented either at the same elevation or ±θ° from it (broken-line rectangles). During the ISI, the participant can make involuntary eye movements in the average range of ±θ° as well. Eye movements produce shifts in retinal and cortical—primary visual cortex (V1)—activations associated with the stimuli (right, broken-line ovals; red spot for activations to the reference stimulus). (B) Example input images used to train the CNN, with or without amplitude modulations (bottom and top rows, respectively). Two leftmost panels show an example of stimulus and movement activity pattern in isolation (not used for training). The vertical rectangle represents activations linked to eye movements; the horizontal rectangle represents retinotopic activations to target stimuli presented at one of the three possible locations in visual space (s1, s2, s3, true labels, white broken-line rectangles in retinotopic space). Corresponding retinotopic activations can occur in one of five possible image (cortical) locations, depending on eye movements (Methods). In parentheses, discretized upward or downward eye movements. Top row, s1(0) is identical to s2(+2); similarly, s1(-1) is identical to s2(+1), exemplifying the ambiguity in location classification introduced by eye movements. Gray levels for bottom images in the [-1.3, +1.3] amplitude range; amplitudes are modulated upward or downward (0.2 steps) from a reference 0.5 value depending on saccadic direction. (C) Schematic of mirror-image activations at area borders in retinotopic regions, unique to visual activations, not observed with movement signals. (D) Basic components of the CNN architecture (also see Methods and S1 Fig). (E) The CNN fails to classify the location of the bar stimuli when the amplitude of the movement activations is constant (three left-most panels and panel (B**)**, top). True labels in the titles; predicted locations on the x-axis labels. However, when movement activations are modulated by the magnitude of the eye movements, the CNN can solve the task (three right-most panels and panel (B**)**, bottom). (F) Classification probability for movement signals added as a global scalar at different stages of the convolutional architecture (Methods; S1 Fig; error bars, s.d., n = 20 network initializations).

Fixational eye movements, apart from being irrepressible and not consciously perceived, introduce a critical “degeneracy problem” in the visual system. To demonstrate this concept, we simulated a classical psychophysical experiment that exemplified the potential disruptive influence of fixational eye movements for visual perception. In this task, which was conducted in darkness and akin to a delayed Vernier acuity task [60,61], a participant is asked to report whether the location of a flashed luminance bar (test stimulus) is above or below a previously seen—but currently not shown—reference location (reference stimulus, Fig 1A). If an involuntary eye movement occurs in darkness between the presentation of the reference stimulus and test stimulus, the target location will often be misjudged with an error proportional to the retinal displacement caused by the eye movement. This indicates that the perceived location depends on the retinal offset in a cranio-centric reference frame (Fig 1A) [62] (reviewed by [36]).

We trained a CNN in a simplified version of this task (Methods, S1 Table). The CNN was required to classify the location of a test stimulus presented randomly at one of three possible locations (Fig 1B). As inputs, we used image stimuli that mimicked retinotopically organized responses in the visual cortex (e.g., in areas V1 and V2; Fig 1C). We also added an activity pattern—dissimilar to the visual stimuli—representing a motor component associated with eye movements [37] (corollary discharge signal; Fig 1B) and as observed experimentally [63–65]. The dissimilarity of the visuo-motor activations holds as a rule: visual stimuli activate posterior visual cortices according to retinotopic mapping, with characteristic mirror-image activations across area borders [66] (e.g., V1–V2, Fig 1C). However, movement-related activations do not abide by these principles [63,67–69]. This dissimilarity was here implemented with movement activations having a different localization and shape relative to visual responses, as observed, for example, in the rodent visual cortex [70]. Retinal displacements caused by eye movements were implemented as shifts in retinotopic activations. Importantly, the size of the shifts was within a range commensurate with the separation among the three locations (angle θ in Fig 1B). This shift introduces a critical ambiguity in the task that makes it impossible to uniquely determine the stimulus location from input activations. For example, an input activation associated with the top-most elevation stimulus could also be elicited by a medium-elevation stimulus after a downward ocular shift (Fig 1B, top panels). Consequently, when trained in this task, the CNN could not discriminate target locations and performance was at chance level (33% correct) (Fig 1D and 1E, left panels). “Failure to classify” occurred when the amplitude of the corollary discharge signal was held constant for all eye movement magnitudes. However, when the amplitude was proportional to the magnitude of the eye movement, as also observed experimentally, the network was able to easily learn the task (Fig 1E, right panels). This is because (1) movement signals carried information to break the ambiguity (degeneracy) between stimulus location and eye movements, and (2) movement activations could not be confused with activity induced by a visual stimulus (non-retinotopic activations). It must be noted that, in the psychophysical experiment as well, when eye movements occurred during the stimulus presentation (i.e., when movement information was made available concurrently with visual signals), localization errors were negligible [61]. Therefore, although in the psychophysical experiment movement signals were always available to brain networks, it was the temporal dissociation between movement and visual inputs during dark periods that led to perceptual errors.

#### 1.1 Classification performance depends on the target layer of eye movement signals

Next, we determined whether the pattern dissimilarity between visual and movement inputs was necessary for successful classification. Alternatively, a *global* (spatially unpatterned) signal carrying movement information added deeper into the hierarchy might suffice. This approach would be equivalent to corollary discharge signals activating higher visual areas (e.g., deeper along the visual stream) in which retinotopy is largely lost and visual responses can still be modulated by movement-related inputs [71]. As implemented earlier, we activated the retinotopic input layer using bar stimuli (with locations degenerate relative to eye movements) and added a global movement signal of amplitude that was proportional to the movement size, either after the second or third convolutional layers (S1 Fig; Methods). To ensure that the network performance was not affected by response saturation caused by the added input, we included a normalization layer immediately after the convolutional one (Methods). With movement inputs deeper in the convolutional architecture, the network could perform above chance, but performance was lower than when movement activations were included in the retinotopic input layer (Fig 1F). This result indicates that when movement information is provided as a global scalar to all neurons deep in the hierarchy, the network can use this information to support a localization invariance relative to retinal displacements; however, the classification can be even more accurate when the signal is provided as an activity pattern in retinotopic visual areas. This result could relate to the experimental observation that motor-related signals are observed also early in the hierarchy of visual areas [72].

#### 1.2 Visual networks activations caused by movements do not induce percepts

We used this computational framework to address a related well-known phenomenon linked to the emergence of visual percepts, rather than to their stability. From the perspective of visual neurons that are activated by movement signals, these cells *agnostically* propagate action potentials down the visual stream, as they would typically do after visual stimulation. These activations can be significant in terms of amplitude and the extent of the areas recruited, even in complete darkness [73,74]. However, neural circuits do not interpret this activity as visual; therefore, no sensory percept is elicited. On the contrary, a rather focal activation of a few visual neurons induced by external stimulation (e.g., electrical stimulation) reliably produces visual percepts (phosphenes) [75]. Although several mechanisms likely explain this difference, we show that simple pattern classification can be a contributing factor. We trained a CNN to classify both visual stimuli and movement inputs implemented as activity patterns that are dissimilar from each other and from visual stimuli (S3 Fig). Learning this task is trivial for CNN models such as those used here (requiring expedients to avoid immediate overfitting issues, Methods). We then tested the network with untrained patterns that mimic small focal cortical activations. The network produced significant probabilities in all output channels, visual and motor, thereby reflecting a large degree of classification uncertainty (S3 Fig). This result follows from the focal activation being largely dissimilar from any trained pattern, either motor or visual. This simple observation may link to phosphene induction: although the visual system is trained (developmentally and/or evolutionarily) to accurately classify movement and visual activations, untrained stimulations—such as those produced by an external electrical pulse—can be partially misclassified as visual (see Discussion).

In sum, in CNN simulations, signals that represent corollary discharges during fixational eye movements can help solving a localization-assignment problem associated with a critical perceptual degeneracy that, in our operational definition, ensures perceptual stability.

### 2. Visual stability during saccadic eye movements

Can this stability principle—that is, a solution to a localization-assignment problem aided by fixational eye movements—be extended to more general eye movements (e.g., saccades) and for stimuli other than simple luminance bars? Answering this question in the context of natural objects implies examining whether corollary discharge signals support the extraction of object-defining features for image classification, invariantly relative to eye movements.

#### 2.1 A general CNN framework for image classification

We considered a similar CNN architecture as earlier, and trained networks to classify a set of natural images from the CIFAR-10 database [76]. This database consists of 10 classes of images with 6,000 examples per class, which adds up to a total of 60,000 images. We modified this database in two ways: first we used black-and-white (BW) images instead of colored ones. This reduced the information available to the network and with that the maximum achievable classification accuracy. Second, we introduced random image shifts to mimic saccadic eye movements—similar to a “data augmentation” procedure [77]. These image shifts could artefactually break the spatial coherence of objects or features in the images—for example, by cropping objects. Therefore, we added a frame (random noise) around the images and implemented saccadic shifts as x-y displacements of the entire image relative to the noisy frame (S4 Fig). For a given image and saccadic shift, the noisy frame introduced an additional axis of variability that the network had to factor-out for accurate classification. Before training with saccadic shifts, we verified that the CNN could be trained with the novel BW framed, but unshifted, images (S5A Fig). Indeed, the network could reach approximately 60% cross-validated classification accuracy (58% ± 1, s.d., n = 5 networks), which is well above the 10% chance level.

#### 2.2 Receptive field properties and responses to simple stimuli

We visualized the preferred features of neurons in CNNs trained with the modified database using the DeepDream response-maximization approach [78]. This analysis indicated that neurons in early layers developed circular receptive fields (RFs) reminiscent of retinal ganglion and LGN cells; in deeper layers, they resembled oriented RFs akin to both simple and complex cells (S5 Fig), as previously reported, e.g., [59,79–82]. Paralleling stimulation protocols typically used in experimental recordings in visual areas, we then examined CNNs responses to oriented gratings. Since CNNs primarily minimized the cost function for the classification of a set of natural images (with <1% of grating stimuli in the training set for this group of simulations), their overall ability to classify grating stimuli was poor and highly variable. However, when examining the neural responses across layers, we found that especially in early layers, neuronal populations could almost perfectly decode stimulus orientations (linear-discriminant analysis, LDA; S5 Fig). This result indicated that while aiming to minimize a cost function for natural images, neuron RFs evolved so that population-level neural decoding of simple grating stimuli could attain accuracy levels far higher than those of the entire network. Notably, a similar observation has been recently made in a study that compared neural decoding in mouse V1 with behavioral discriminability [83] (see also Discussion).

#### 2.3 Eye movement signals improve classification

**Fig 2:**
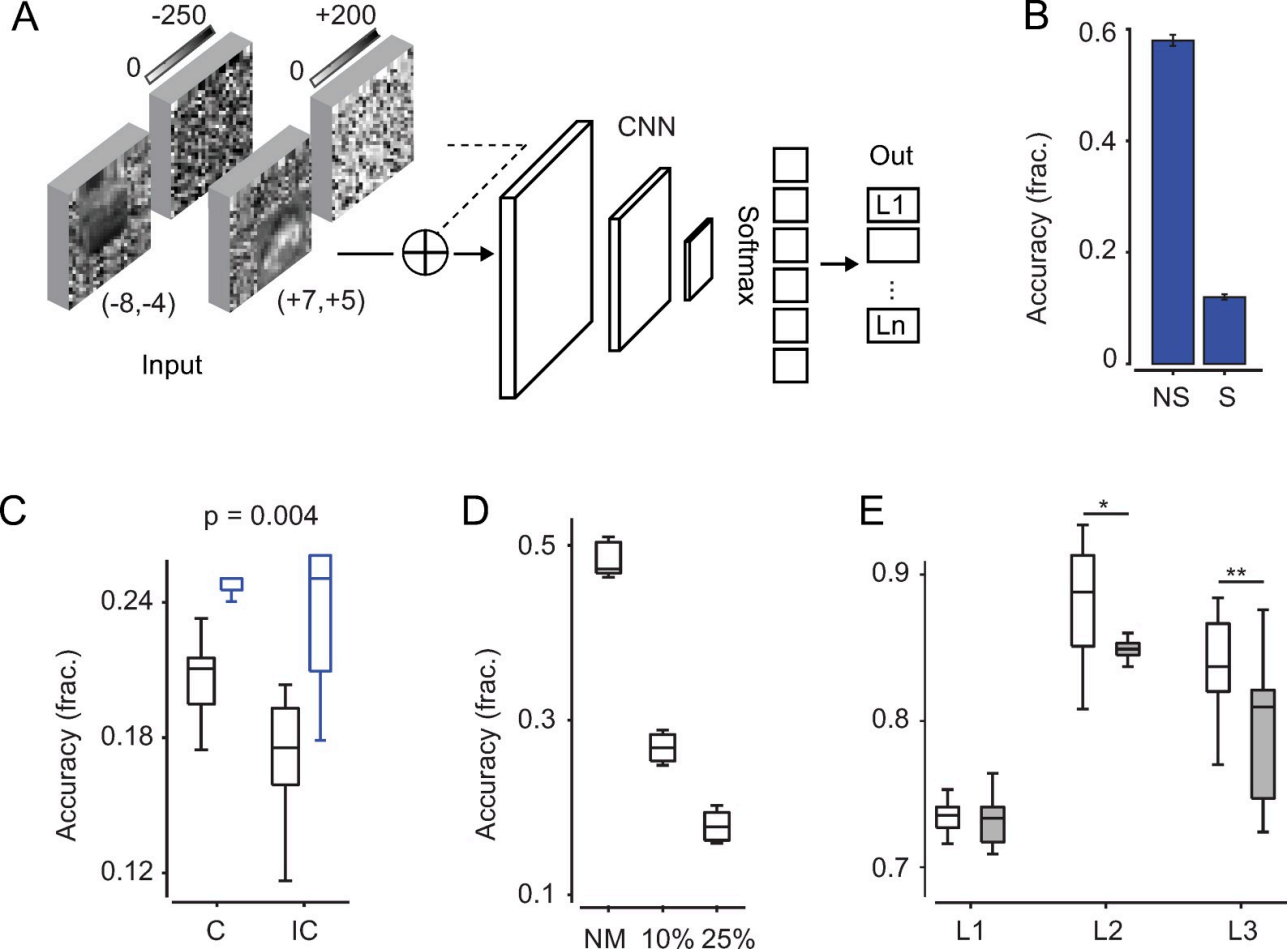
Eye movements support classification accuracy of image categories and saccadic directions. (A) Schematic of the network architecture. Noise-framed and shifted CIFAR-10 images were provided at the image-input layer. Value pairs in parentheses indicate the numeric amplitudes used for horizontal and vertical image shifts, relative to the center: negative values for top-left shifts, positive for bottom-right ones. Image amplitudes were in the [0,255] range. Movement inputs were also provided as images (pixel noise) with amplitude values scaled to match (with sign) the amplitude range of the images (congruent condition). In the incongruent condition, movement amplitudes were randomly assigned to shifted images. A feature layer was added to the image layer before conv-2, or other convolutional layers in control simulations. (B) Classification performance (accuracy) of CNNs trained with no saccadic shifts and tested also with no saccadic shifts (“NS” label) or with saccadic shifts (“S” label). (C) Performance of CNNs when trained with congruent and incongruent movement inputs (black boxes). Only networks that reached above chance-level performance (10%) were compared. Blue boxes for quadrupled number of training epochs. (D) Classification accuracy when training with saccadic shifts and varying amplitudes of incongruent movement inputs: zero amplitude (NM, no movements), 10% and 25% of the input-image amplitude, with 25% being the typical scaling value used in main simulations (Methods). (E) SVM classification accuracy of saccadic directions from channels’ activations in different layers; the SVM classifier was used to separate 3 conditions: 1st quadrant, 3rd quadrant, and 2nd or 4th quadrant (Methods, S2 Fig). Empty boxes for networks performing image classification above chance level (n = 16); filled gray boxes for networks at chance level (n = 14); p = 0.01 and p = 0.006 (*, **) Wilcoxon rank sum test.

Having established that CNNs could be trained with unshifted images from the modified CIFAR-10 database, we then introduced saccadic eye movements. A CNN trained with no image shifts and tested with simulated saccades, performed at a near-chance level (Fig 2A and 2B, 12% ± 0.5%, n = 5). However, biological visual networks are not “developmentally trained” with still visual inputs, and corollary discharge signals continuously activate visual networks likely playing a critical computational role for perception during saccadic shifts. Therefore, we trained CNNs with saccadic shifts in the input images as well as with movement signals, comparing two conditions: with movement amplitudes that are informative about the eye movement vector [64] (hereafter referred to as *congruent* condition) or having amplitude values unrelated to the shift vector (random assignments, *incongruent* condition). Movement signals were added as a second input (feature) layer (Figs 2A and S1) and implemented as spatially unstructured noisy images—thereby being largely dissimilar from visual inputs—whose values either (partially) reflected the vector components used for the saccadic shift or had random values (S2 Fig). Optimization parameters were approximately set so that the network could (1) reach above-chance performance levels and (2) have performance levels that could differentiate between the two training conditions (congruent vs incongruent, Methods). We found that in congruent conditions CNNs classification was more frequently above chance level—relative to different initial conditions—and when above chance, the classification accuracy was, on average, higher than that in incongruent conditions (53% and 36% of the networks, n = 30 networks, reached above-chance performance in congruent and incongruent conditions, respectively, with classification accuracy 21% ± 2% and 17% ± 3%; p = 0.004, t-test, n = 16 and n = 11 networks; Fig 2C). An inspection of the confusion matrices revealed that when the classification accuracy was approximately 20%, the network could, on average, accurately distinguish between the various image classes, but with a fair degree of variability (S6 Fig). Instead, networks with above-chance classification, but below 20%, typically overclassified a sub-set of classes. At chance level, the sub-set could be as small as a single class. Further, injecting movement signals at later stages reduced the probability of successful classification in all trained networks to chance level (10% for layers 2 and 3 in 10 out of 10 networks).

Congruent movement inputs improved classification by accelerating the training and making the network more robust relative to input noise. Indeed, when quadrupling the training epochs, performance reached similar levels in both congruent and incongruent conditions (Fig 2C, blue boxes, 24.7% ± 0.5% and 23% ± 3%, for congruent and incongruent conditions, n = 10), indicating that networks needed fewer examples to reach above-chance performance levels with congruent movement inputs. Further, training CNNs with saccadic shifts but no movement inputs allowed for large classification accuracy (48% ± 2%, n = 5, with and without noisy image frame). The accuracy decreased, but remained well-above chance level, when incongruent movements with smaller amplitude were added (60% smaller than in main simulations, Fig 2D, 26% ± 3%, n = 5). This indicates that movement inputs, in general, decreased network performance, affecting the network as pixel-level input “noise”, but performance degradation was significantly reduced in congruent conditions (Fig 2C).

Finally, although the networks were trained to classify image categories, they could also classify the direction of saccadic displacements. We binned saccadic movements into saccadic-direction groups and used a linear SVM classifier trained on the channels activations. In all layers, classification accuracy was above chance level, exceeding 80% accuracy in layer 2 and 3. In deeper layers, networks with object-classification accuracy above chance level had significantly higher decoding accuracy for saccadic directions (L1, n.s., p = 0.4; L2, p = 0.01; L3, p = 0.006; Fig 2E).

Overall, these results indicated that movement signals—whose activity patterns were largely dissimilar from those of visual inputs—when carrying information regarding the saccadic vector, enabled a more successful extraction of image-defining features for accurate classification, invariantly relative to saccadic shifts, accelerating the learning process and making the networks more robust relative to input noise. Furthermore, movement information was better encoded in networks with higher classification accuracy, possibly reflecting their key role in building a classification invariance for saccadic displacements.

#### 2.4 The network architecture affects classification performance

Shallower and deeper architectures significantly affected the CNN performance. When using AlexNet (with 5 convolutional layers, hence deeper than our basic CNN with 3 convolutional layers, S1 Fig), trained with unshifted CIFAR-10 images (BW and rescaled as in the main simulations) and with no movement inputs, average performance was rather poor, 18% ± 3% (n = 3, and n = 2 at 10% chance level). The network could not be trained above chance level when introducing image shifts, regardless of movement inputs (n = 5 initializations with movement inputs and n = 5 without). This was observed even when relaxing the early stopping constraint, quadrupling the number of training epochs. A shallower architecture was tested as well, using a CNN with two convolutional layers. When trained with only unshifted CIFAR-10 images (BW and rescaled as in the main simulations and with no movement inputs) average performance was 40% ± 2% (n = 5). When trained with shifted CIFAR-10 images but no movements, performance was at chance level (n = 5), but when trained with saccadic shifts of reduced size (50% smaller than in main simulations) and still no movement inputs, performance could be above chance level (out of n = 5 initializations, n = 2 at 30% ± 3%, and n = 3 at 10% chance level), indicating that the amplitude of the saccadic shifts significantly affected network performance relative to its architecture. Although the network was unable to perform above chance level when both image shifts and movement inputs were introduced (n = 5 all at 10%), significantly reducing both amplitudes (saccadic shifts by 75% and movement amplitudes by 80%) allowed the network to partially learn (30% ± 1%, n = 2, and n = 3 at 10% chance level). In summary, saccadic shifts combined with additive pixel “noise” (movement inputs) of amplitude commensurate to that of the input images degraded the performance of both deeper and shallower architectures, with the amplitude of both these parameters affecting overall performance.

#### 2.5 Eye movement signals enable sharper classification boundaries

**Fig 3:**
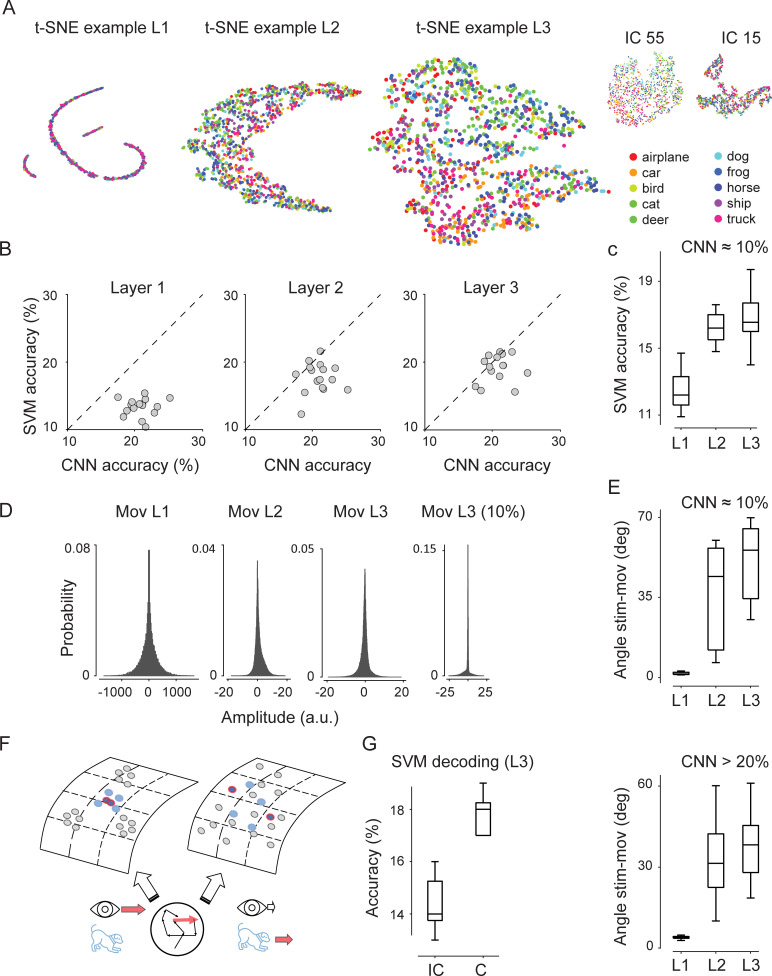
Eye-movement inputs support the clustering of object-class representations. (A) Representative example of t-SNE embedding for visual inputs only, and for a network that attained above chance-level classification performance with saccadic visual shifts and congruent movement inputs. Color hue indicated a spatial gradient for object classes (legend) in layers 2 and 3. Top-right plots, representative networks trained with incongruent movements (IC) and end-stopped at 15 or 55 epochs. (B) Accuracy of SVM models trained on t-SNE representations as a function of the accuracies and layers of CNNs (CNN performance values are constant and defined as the overall output classification). Each point represents a network with a different weight initialization. Only in L3 do the data points cluster near the diagonal, with SVM performance values approximately matching overall CNN performance values. (C) Accuracy of SVM models trained on t-SNE representations across layers (x-axis) for networks that did not exceed 10% chance-level performance. (D) Representative examples of probability distribution amplitudes for channels in different layers in networks that attained above chance-level classification accuracy, with saccadic shifts and congruent movement inputs. Amplitude distributions are computed when testing networks with movement inputs only. Movement inputs have significant non-zero amplitudes at all layers. Rightmost panel (Mov L3 (10%)) for an example network with chance level performance, showing larger zero-amplitude probability in L3 (“gated-away” movement responses). (E) Angular distance between activity axes (modes) across layers in low- (top) and high-performance (bottom) networks. (F) Left, schematic of self- vs external-movement stimulus representations. The movement of a stimulus (blue dog) across the retina (red arrow inside the circle, with black arrows representing continuous fixational eye movements), is caused by a self-generated eye movement (left, long red arrow near the eye), or by the stimulus moving—still in the presence of fixational eye movement (right, medium length red arrow near the dog, and short empty arrow near the eye). Stimulus representations are more clustered during self-generated eye movement with congruent motor-related signals: top-left, blue dots with red contour lines for the example saccadic shift. Gray dots for other stimuli possibly present in the scene. Top right, external movement is linked to less clustered representations. (G) SVM decoding accuracy of L3 activations in networks trained with congruent movements and tested with saccadic image shifts and congruent or incongruent movement inputs (n = 10).

Next, we used a representational analysis to examine how CNNs learned to use movement signals for shift-invariant classification. We considered networks trained with congruent movement signals that reached classification accuracy above chance level. Thereafter, for a given image, we computed a mean response for each channel by averaging activity across its entire receptive field (units). Thus, at each layer, we obtained population responses to example images from different classes and for various saccadic shifts (and framing noise). We visualized the high-dimensional population activity in a 2D manifold using a stochastic-neighbor embedding method (t-SNE, Fig 3A) and then applied a support-vector machine model (SVM, Methods) to classify the identity of the shifted images. We found that across all convolutional layers, SVM accuracy was significantly above chance, with the greatest accuracy at the third convolutional layer (13% ± 1%; 18% ± 1; 19% ± 2%, in layer 1–3, n = 16 networks; Fig 3B). As a control, an SVM model on randomly shuffled labels performed at chance level (10%), as expected. Using an SVM model on PCA representations yielded almost identical results (14% ± 2%; 18% ± 1; 19% ± 2%, in layer 1–3, n = 16 networks). Notably, the SVM model could perform above chance even when the overall network performance was at chance level (Fig 3C), particularly in convolutional layers 2 and 3, with the SVM model on t-SNE representations performing approximately equally well as with PCA (e.g., in L3: 17% vs 16%, respectively). This might simply reflect the early-stopping procedure used to reduce overfitting, with the trainable weights in the fully connected layer not (yet) sufficiently optimized to take advantage of the representational clustering that emerges in deep layers. Further, confusion matrices indicated that a quadratic SVM model trained on t-SNE data correctly distinguished—on average and mainly in L3—among image classes, although with a fair degree of variability. Instead, a linear SVM model trained on PCA data, despite reaching comparable overall performance, tended to overclassify a few specific classes (S7 Fig), thereby suggesting a non-linear representational embedding of the image classes.

Experimental observations made at the local-circuits level [84] and at the multi-area level [70], have shown that visual and motor signals, concurrently activating visual areas, can be represented by distinct low-dimensional activity manifolds, that, at the mesoscale level, are near orthogonal to each other [85]. Therefore, we next examined the propagation of visual and motor signals across layers and the separation of the representations. In networks that did not classify above chance levels movement responses were progressively “gated-away”, almost zeroing movement amplitudes at the last convolutional layer (Fig 3D). This was also reflected in the angular separation of activity axes (modes) [86] for movement and visual responses: in layer 3, the angle between activity axes was larger when the network did not learn and this followed from most units being unresponsive to movement inputs, thereby increasing the dissimilarity between movement and visual activity patterns (51° ± 16° and 38° ± 11°, p = 0.01, n = 16, wo/w learning, respectively; Fig 3E).

In sum, a representational analysis of the high-dimensional space of CNN activations, revealed that training with informative saccadic movements supported the non-linear clustering of population responses into sub-spaces associated with image classes, more prominently at the intermediate and late convolutional layers [87]. The presence of these categorical sub-spaces likely reflected a learned invariance for saccadic shifts.

Results from the t-SNE analysis suggest that in networks successfully trained with corollary discharge signals (congruent condition), movement signals might support a more “clustered” stimulus representation than with (untrained) external motion, simulated as incongruent movement signals. That is, in the absence of external motion but in the presence of eye movements, a shift of a stimulus across the retina would cause a “small” representational shift (Fig 3F) supported by congruent corollary discharge signals. Instead, if the same retinal activation was caused by external motion—still in the presence of irrepressible eye movements (e.g., fixational movements)—corollary discharge signals would be incongruent relative to the retinal shift vector and, not being part of the training set, they should produce a “larger” shift of the stimulus in the same representational space (Fig 3F). We tested networks trained in the congruent condition with image shifts and movement inputs that were either congruent (self-motion) or incongruent (untrained external motion; note that in the t-SNE analysis of Fig 3A and 3B only visual inputs were provided to the network). As expected, when tested with incongruent movements, overall classification accuracy decreased (14% ± 4%, n = 30). We then examined CNN activations in different layers in a reduced PCA representational space (20 dimensions) and used an SVM decoder to estimate the clustering, or mixing of the representations in these two conditions. We found that in the deep layer L3 the decoder could more accurately separate the image classes when movement inputs were congruent than in incongruent conditions (Fig 3G, p = 0.001, Wilcoxon test, n = 10), with no significant differences in earlier layers. Therefore, in networks trained with congruent corollary discharges, not only visual inputs, but also co-propagating visual and motor signals produced more stable (clustered) network activations for different image categories during self-motion than in the presence of (untrained) external motion (see Discussion for the interpretation of external motion simulations).

#### 2.6 Saccadic modulations of neural responses

**Fig 4:**
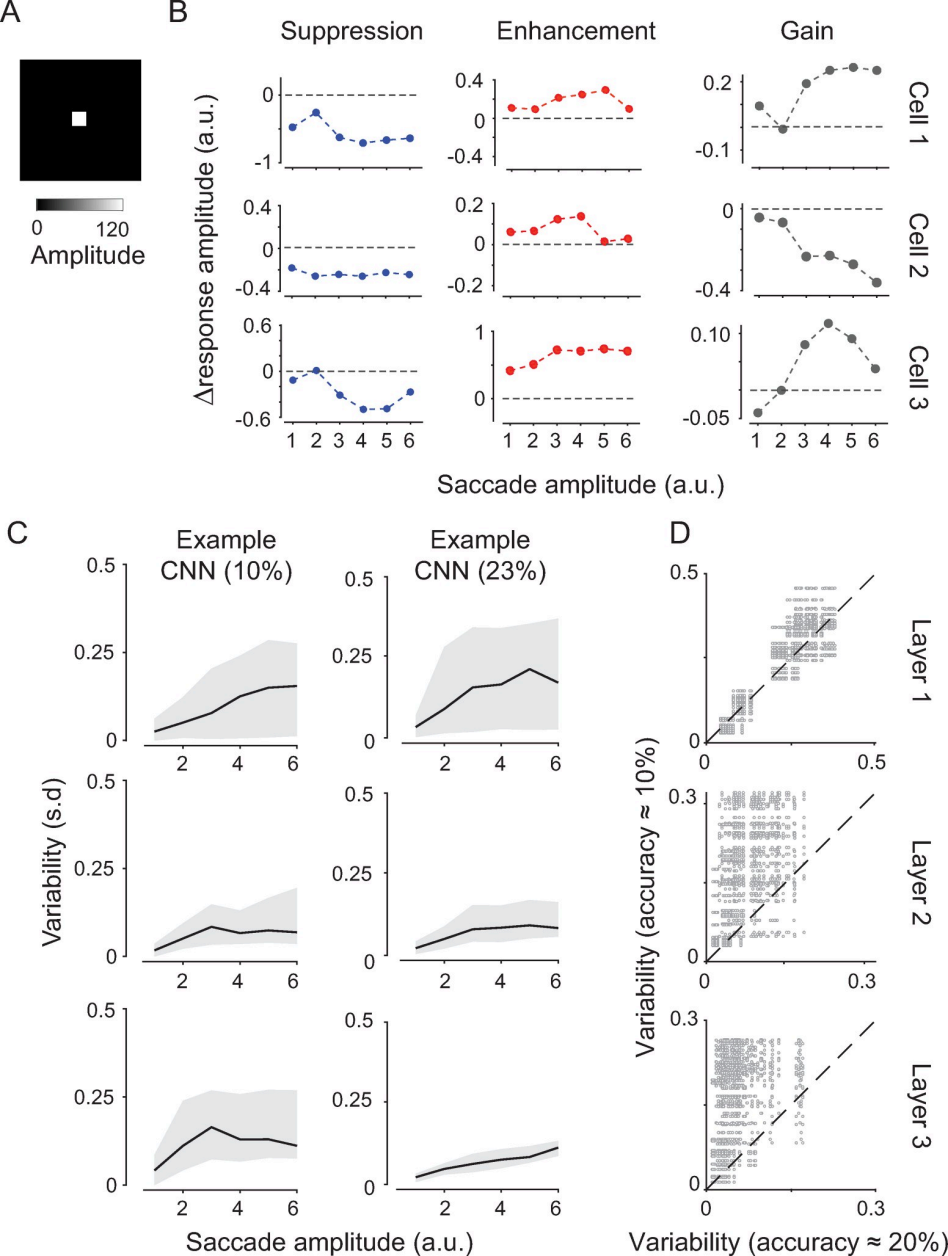
Saccadic modulation of single cell activations. (A) Test stimulus used to measure cell activations in the presence or absence of saccadic shifts. (B) Neurons showed diverse response modulations by saccadic inputs—that is, response change w/wo movement inputs (representative examples). For some neurons, responses were either overall suppressed or enhanced, largely independently from the amplitude of movement inputs (first two columns). For other neurons (third column), the response change was proportional to the movement amplitude; “gain” is used in reference to gain fields. Smallest to largest saccades grouped into six linearly spaced amplitude bins (Methods). (C), Variability in saccadic modulation across cells (s.d.) as a function of saccade size (x-axis) in different layers (rows) and for networks performing at chance level (left column) or above (right column). The solid line indicates the mean; gray band indicates the 5th-95th confidence interval (CI). (D) Scatter plot of maximum variability values (CI width, Methods) between networks at and above chance-level performance. Each dot refers to an example movement input.

Finally, we examined whether we could identify response signatures that reflected the network’s ability to utilize movement signals for shift-invariant classification at the level of individual channels. As typically done in electrophysiological studies of response modulations by eye movements [4], we tested trained networks with a luminance stimulus (Fig 4A) and measured responses across layers, first without saccadic inputs and then with saccadic inputs of different amplitudes. Since these CNN simulations did not explicitly include time, computed activations could be viewed as analogous to taking peri-saccadic time averages of neural responses. By comparing responses across the two conditions, we found a variety of modulations of the stimulus-evoked response, which are reminiscent of similar effects reported in biological neurons (e.g., [64,88]) (Fig 4B). Specifically, as observed during saccadic suppression of the visual percept [89,90], we found that several CNN responses were suppressed in the presence of saccadic signals, approximately equally across saccade magnitudes. In contrast, other channels enhanced their response amplitude, as also observed experimentally in (post) saccadic response enhancement [91,92]. Further, several responses were modulated by the amplitude of the saccade, with larger amplitudes amplifying the suppressive or enhancing effects (Fig 4B). This phenomenon also has a biological counterpart, which is referred to as “gain fields” [42,65,93,94], where the same retinotopic stimulation elicits responses of different amplitudes depending on the angle of gaze. Although the type of modulation was largely layer-independent, the across-channel variability in saccadic modulations was layer-dependent, with a sharp decrease in variability from the input to the output layers (Fig 4C). Notably, this feature was characteristic of trained networks; although networks with chance-level classification accuracy also exhibited similar saccadic modulations, the across-channels response variability did not decrease in deeper layers (Fig 4D). The decrease in variability in late layers may be related to the emergence of categorical representations revealed by t-SNE analysis of networks performing above chance level.

In summary, in successfully trained networks, saccadic inputs affected the stimulus-evoked response with response suppression, enhancement, and amplification of these effects depending on saccade size, as similarly observed in biological visual neurons. The hallmark of trained networks was a decrease in the across-channels variability of these modulations along the convolutional architecture, accompanied by the significant image-feature clustering highlighted in the t-SNE-SVM analysis.

## Discussion

In this study, we adopted an operational definition for perceptual stability as the activity state of a computational system that classifies features in a visual scene, invariantly relative to image displacements resulting from eye movements, while integrating motor-related signals informative of retinal shifts. In this operational definition, the categorical classification output might relate to perception-related decision processes, such as action selection and execution; however, perception is rather associated with activity states distributed across network layers, and therefore continuous and variable, reflecting the great diversity between exemplars of the same category. This operational definition of visual perception blurs the boundaries between sensation and perception [95], with early sensory (retinal) layers being as much “perceptual” as the deep ones. By adopting this framework, we related the stability of visual perception to an optimization problem with the aim of acquiring a classification invariance relative to retinal displacements. We discussed this invariance in relation to fixational and saccadic eye movements. For fixational eye movements, we considered a similar task to that of [61], which cleverly demonstrated the disruptive perceptual effects of small ocular movements. When visual and movement-related neural signals are temporally decoupled (in darkness), subjects misperceived the location of probes having angular separations commensurate to the ocular shifts, as predicted by retinotopy. However, movement-related activations, when coupled with the stimulus can be used for an accurate cranio-centric localization of the probe during fixational eye movements, thereby breaking a degeneracy in the stimulus localization, as shown behaviorally by [61] as well as here in CNN simulations. Generalizing from small luminance bars to objects, these results suggested that corollary discharge signals may support the correct identification of object-defining features in the presence of unperceived fixational ocular movements, and, according to our operational definition, achieve stable perception. We directly tested for this corollary, and extended its scope to larger eye movements, by examining CNNs classification accuracy for natural images in the presence of image shifts that mimicked saccadic eye movements. We found that movement signals, informative of the saccadic shifts, improved classification by speeding up the training, by making the network more robust relative to input noise, and by supporting the representational clustering of object categories, more significantly in deeper layers.

### Limitations of CNN as mechanistic models of the visual system

CNNs are increasingly popular representational and quantitative models of the hierarchically organized visual system for core object recognition [58,59,96], with pioneering studies explicitly inspired by biological visual architectures [97]. As implementation-level, mechanistic models, some of their characteristic features are often considered at odd with biology [59,98], such as weight sharing and convolutional operations. However, these differences are hardly unreconcilable with biology. Weight sharing, for example, is computationally convenient but not indispensable, with the key network operation on input images grounded on the filtering operations of neurons across channel, constraining feature maps. In biology, considering, for example, the processing of stimulus orientations, neurons with localized receptive fields “pool” from localized synaptic patterns, possibly based on random connectivity weights [99] to create filters having similar characteristics that cluster in cortical domains, and collectively processing image features across the entire visual field. In response to a localized oriented stimulus a “feature map” is activated, described as a standing wave of activity [100], which recruits neurons in orientation domains tuned to the stimulus orientation. Concerning the convolution operation, CNNs implement it by moving filters along the input and computing the dot product of the weights and the input. In biology, neurons that take a dot product of the incoming firing rates with the receiving synaptic weights to obtain the output activation have been described in the context of “dot product decoding”, introduced as a biologically plausible code in neuronal circuits [101].

### Novelty relative to previous modeling works

CNNs can easily learn translational invariances without the need of any additional input signal (as briefly reviewed in the Introduction). Indeed, when training CNNs with no saccadic shifts and without movement inputs, performance was largely above chance level, with the CIFAR-10 exemplars representing a given object-class under various translations, rotations, and size changes. However, these *intrinsic* invariances (often built-in in the training process using data augmentation methods), are not explicitly representative of the perceptual stability phenomenon; as exemplified by fixational eye movements, the same retinal activation (image shift) would elicit a percept of movement when the activation is due to a movement in the environment, or an unchanged visual percept when the activation is due to a self-generated movement. Intrinsic invariances do not distinguish between these two conditions. Instead, corollary discharge signals play a critical role in this distinction: they continuously activate visual network (e.g., fixational eye movements cannot be voluntarily suppressed) and the information they carry is used to suppress only the percept of self-generated movements. In our simulations, despite learning *intrinsic* translational invariance from the CIFAR-10 exemplars, networks without movement inputs, trained with no saccadic shifts, and tested with saccadic shifts performed near chance level. This is not surprising when considering that the statistical properties of the simulated saccadic shifts (e.g., large translations with noisy fitted frames) were largely dissimilar from those of the exemplars in the CIFAR database. This scenario as well can be linked to biological networks, where the statistics of retinal activations induced by saccadic eye movements do not necessarily match those induced by movements in the external world [102]. In the latter condition, stable object recognition in the presence of external movements may be achieved directly from the movement statistics, without the need of corollary discharge signals, as suggested, for example, in unsupervised temporal learning models and slow feature analysis models. This observation helps interpreting results in Fig 3, in which SVM classification accuracy in L3 decreased when networks trained with congruent movement inputs were tested with saccadic shifts and incongruent movements to simulate external motion. This result support the interpretation that corollary discharge signals aid representational clustering in deep layers and it does not imply than external motion leads to poorer classification. Indeed, CNNs were not trained with incongruent movements, and when networks were trained with such movements they could learn above chance level but it took longer, with the learning relying on mechanisms other than the corollary discharges, for example those learned directly from the movement statistics, both intrinsic and saccadic related.

It should also be noted that besides their role in perceptual stability, corollary discharges activate visual networks possibly for several other computational goals in sensorimotor processing, such as predictive inference computations in dynamic agent-environment interactions [24,103–105]. Therefore, in our simulations we considered corollary discharges always activating the network and contrasted two conditions: when they were informative of the self-generated movement and when they were not.

As briefly reviewed in the Introduction, several previous modeling works have focused on peri-saccadic phenomena related to how spatial information is updated, retained, or inferred during eye movements, including peri-saccadic perceptual distortions and errors. What all these studies have in common is the primary focus on peri-saccadic localization phenomena, with the underlying assumption that by learning localization computations during eye movements it will then be possible to gain a better understanding of how brain networks use those computations to achieve stable perception. Here, we adopted a different conceptual and computational framework. Instead of having a primary focus on a localization (“where”) question, we focused on a classification (“what”) problem in the presence of eye movements. The cost function used in our simulations with natural images minimized a classification error for object identity, and not for its location, with “how” this minimization was achieved relating to the acquisition of invariances for spatial transformations.

### Dorsal and ventral streams

Computationally, we adopted CNNs as models of the cortical visual hierarchy. Indeed, CNNs represent state-of-the-art quantitative models of the mammalian visual system, most prominently of the ventral (“what”) visual stream [58,59,96,106,107]. However, the dorsal (“where”) visual stream is also characterized by a hierarchical architecture and has been traditionally associated with perceptual stabilization during movements [108,109]. The architectural design and optimization process adopted in our simulations has “hybridized” dorsal and ventral streams of information processing [110,111]. Indeed, we used an image classifier (i.e., a “what” type network) that also processed movement inputs (“where” information) to extract image-defining features, invariantly relative to saccadic shifts. Biologically, this can be interpreted as “what” type of information processing along the ventral visual stream with the integration of movement information from the dorsal stream to achieve movement-invariant classification [64]. Alternatively (or concurrently), the dorsal stream might also integrate “what” information from the ventral stream to support movement-invariant classification. How distinctively these streams process and share information and whether they might eventually “merge” information onto a perceptual output stage remains a matter of debate [112]. Our mechanistic framework provides a convenient testbed for these investigations, and future research could examine the representational similarity between neurons along the dorsal and ventral streams during saccadic eye movements and networks trained with corollary discharge signals to localize features in visual scenes, invariantly relative to image displacements [113].

### The geometry of visuomotor representations

By using CNNs as representation-level models, it is possible to analyze the geometry of the neural representations in artificial and biological networks and their similarities at various stages along the visual hierarchy [114]. Here, we performed some simple analyses in this direction. Together, results based on t-SNE-SVM classification and decoding analysis, the angular distances between activity axes, and the decoding of saccadic directions, suggest a simple representational framework: in networks that successfully used movement information for image classification, movement and visual activations defined disjoint activity sub-spaces with also shared dimensions that could lead to increases in neural activity in response to both visual and movement inputs. During training, the shared dimensions allowed movement information to aid the representational clustering of natural images in the visual subspace (t-SNE analysis) and were reflected in the mixed visuomotor selectivity typical of most neurons. Representational clustering in t-SNE space is reminiscent of the concept of object manifold [87], where various transformations of the same object are represented by activity states (here t-SNE points) that are clustered together in low-dimensional activation sub-spaces. Experimental evidence supporting disjoint activity sub-spaces with also shared dimensions comes from works on the integration of visual and motor signals using high-resolution videography combined with neural recordings from cortical visual areas [84], and from a recent study examining eye movements and their integration with visual inputs, finding indeed separate activity manifolds associated with visual and motor variables together with shared low-dimensional co-variability subspaces [70].

### Shift-vector information in movement signals

In our simulations we made two assumptions related to the properties of movement signals: (1) that movement inputs carried (approximate) information related to the saccadic shift-vector and (2) that movement inputs could potentially propagate “ungated” along the visual stream. Indeed, experimental work has shown that even as early as in the V1 “eye tracker” information can be extracted from neural signals to accurately reconstruct the direction and gaze and, therefore, the location of objects in a craniocentric reference frame [64]. Further, psychophysical experiments have confirmed that visual information is not gated away at specific layers in visual streams but remains available to perceptually relevant visual circuits, for example, permitting learning-mediated improvements of perceptual judgements during saccades [102,115].

The spatial structure of movement signals was implemented either as a coherent pattern in fixational eye movement simulations or as scaled salt-and-pepper noise in simulations with saccades. The first choice was motivated by the need to classify movement patterns as well, which was not required in the latter case. Irrespective of the specific implementation, in both cases, the key requirement was that movement signals had spatially distinct signatures from image inputs; under this constraint, movement inputs were able to aid the output classification.

### Layer specificity and architecture

In simulations with luminance bars and natural images, adding movement signals at later stages (layers) along the convolutional architecture lowered the overall network performance. This observation suggests that, in the context of the architectures explored here, it might be advantageous for visual stability to process movement signals early in the hierarchy, as also observed experimentally [64,116]. This observation may not be simply explained by a stronger computational machinery (i.e., more downstream convolutional layers) when adding movement inputs in early layers; indeed, depending on the task at hand, and aside from overfitting issues, simply adding layers does not necessarily correlate with improved network performance [96,117]. We have also partially explored the importance of the network architecture by considering deeper and shallower architectures. Indeed, we found that deeper is not necessarily “better”, and that network depth, saccadic shift size, and amplitude of the motor inputs synergistically modify classification performance. These results suggest that a more extensive approach should be adopted to explore the layer dependence, evolving architectures and performing an architectural search constrained by the objective function described above [118]. This approach is likely to produce more stable and significant results than simply attempting “by hand” a few modifications of the current architecture. This approach might also allow us to separately explore the contributions to classification of pattern similarity between visual and movement activations and the “injection layer” for movement signals along the visual hierarchy. Exploring these and other hyper-parameters would be best achieved with automatic hyper-parameter optimization methods [119].

### Phosphenes and “off-manifold” activations

In simulations with fixational eye movements, we examined the implications of pattern separability in visuomotor integration in the context of phosphene perception. Externally induced activations of rather small neuronal ensembles can produce a visual percept (phosphene); however, large-scale activations of millions of neurons along visual streams induced by body movements (extra-retinal inputs) do not elicit percepts. We reproduced a much-simplified version of this result based on the simple consideration that electrical stimulation induces “untrained” activity patterns, which, in the manifold terminology, would correspond to off-manifold activity states [87,120]. The underlying assumption for this observation is that biological visual networks have been developmentally (and evolutionarily) trained to distinctively classify self-body-movement signals from retinal inputs along sensory streams. This simple “classification-based” interpretation agrees with the reported separation of visual and movement representations in CNNs, and observed in multi-area activations in biological cortical networks [70]. This interpretational framework may also explain why responses evoked by electrical stimulation of the retina are instead interpreted by the visual system as natural stimuli; indeed, these stimuli induce phosphenes that are subjected to saccadic suppression, similar to what occurs with natural stimuli [121], which is not the case for cortical-induced phosphenes. According to our interpretation, this is because artificial activations of retinal ganglion cells is barely distinguishable from “trained” natural luminance patterns that activate the retina (i.e., in-manifold patterns). For example, during natural viewing, it is not uncommon that only a small, localized group of photoreceptors is activated in dark conditions by a few pinhole lights in the environment (the stars in the night sky being an obvious example), and both pinhole light and ganglion cells electrical stimulation activate the primary visual cortex according to topographic principles. However, phosphenes induced by electrical stimulation further down the visual stream (e.g., in the occipital lobe) are largely dissimilar from any trained activity pattern—for example, by being associated to non-retinotopic activations (i.e., off-manifold); therefore, they are “misinterpreted” by the visual system and, for example, although perceptually salient, they become immune to saccadic suppression [121]. Together, these observations explain phosphene phenomena in a unified computational framework, where the separate encoding of visuomotor variables is associated to correct output “classification” of these variables [70,84].

### Orientation-discrimination hyperacuity

In basics characterizations of networks trained with natural images we found a near-perfect discrimination of grating orientations from neural activations in convolutional layers, associated with an overall poor classification performance by the network. Computationally, the overall poor classification simply resulted from gratings being a very small sample in the training group; classification obviously improves with a larger proportion in the training set. However, this analysis focused on the link between a network’s objective function and the encoding/decoding properties of its channels at various layers. In optimizing the objective function, channels in a given layer can evolve to possessing receptive fields features such that their classification performance for a specific class of stimuli becomes largely superior to that of the network. We exemplified this aspect in the context of grating stimuli; however, in all generality, using, for example, a DeepDream approach [78], it is possible to select a vast set of complex images that maximally and differentially activate units in a given layer, thereby resulting in high classification accuracy provided by that layer, but with rather poor performance by the network, see also [122–124]. It is likely that a biological counterpart of this phenomenon has already been demonstrated. In mice, for example, behavioral orientation discrimination thresholds have been shown to range around a few degrees [125], but cortical recordings in visual areas have revealed a sub-degree precision [83]. In this context, trial-to-trial internal (downstream) “noise” hypothesized to corrupt signal processing and behavioral accuracy might rather simply reflect appropriate signal transformation according to an objective function optimized for natural image statistics.

### Suppression, enhancement, gain fields: Limitations of our approach

The effect of saccadic inputs on the stimulus-evoked response was suggestive of biological phenomena, such as saccadic suppression (and its neural correlates), post-saccadic response enhancement, and gain fields. These qualitative parallels must be carefully examined in view of the obvious limitations of our simulation environment. For example, saccadic suppression—behaviorally defined (e.g., [126])—has a suppressive neural signature that precedes the saccade by several tens of milliseconds, with a spatial localization in dorsal motion-sensitive areas, such as areas MT, MST, and VIP [71,89,102,127] as well as in the superior colliculus [128]. Instead, post-saccadic response enhancement is observed at a short latency after the suppression and across similar regions [91]. Further, gain fields reflect a neural modulation by gaze angle to a retinotopic stimulation [42,43]; hence, saccadic movements per-se are not “dynamically” involved in this phenomenon. However, it must be noted that the position of rest of the eyes is divergent (exotropic) [129,130]; thus, to maintain an angle of gaze, stabilizing and accommodative muscular responses are necessary [130], with corollary discharge signals linked to these muscle activations likely to vary with gaze-angle size (distance from resting position) and broadcasted along sensory streams, just as any other corollary discharge signal [24]. The temporal characteristics of these phenomena indicate the differential recruitment of circuits and mechanisms over time, which cannot be simulated in the context of CNN architectures, critically missing dynamic components. The importance of the temporal dynamics is apparent also from strong peri-saccadic psychophysical phenomena; for instance, brief flashed stimuli are mislocalized starting from before the saccade onset [32,36,38] and, in regions such as LIP, eye movement information can be updated quite late after saccade execution [131]. For this, recurrent neural networks (RNNs) might be a more apt modeling framework. However, qualitatively, the observation of these phenomena in CNN simulations suggests that these response features can naturally emerge in architectures that integrate visual and motor signals. At a more general level, this reflects the rather unsurprising observation that when CNNs integrate two input signals, these signals, to a first approximation, can influence each other in the form of signal enhancement, suppression, and amplitude-dependent modulations of the two. RNNs might add to this simple observation by revealing that a peri-saccadic modulation of these phenomena can contribute to the network classification accuracy in a shift-invariant manner. In addition, what was less expected was the finding that when the network learned to use movement signals to extract image-defining features, the neuron-to-neuron variability decreased along the hierarchy, along with increased clustering of t-SNE representations and SVM classification accuracy. These are experimentally testable predictions for future studies that focus on the representational encoding of natural images by neuronal populations along the visual stream in the presence of saccadic eye movements.

In conclusion, our results mechanistically explain the computational advantage of having corollary discharge signals for sharing neural circuits with visual inputs along the visual hierarchy in order to achieve perceptual stability. Because movement-related activations have also been observed extensively in non-sensory regions [132] this study provides a more general computational framework to examine fundamental integration principles of movement signals with local computations during complex animal-environment interactions.

## Methods

All simulations were performed using MATLAB Deep Learning and Neural Network toolboxes (The Mathworks, Inc., 2020).

### Network architectures

CNN architectures are presented in S1 Fig and S1 Table. All four main architectures had three convolutional layers with 3 × 3 filter sizes—with weight sharing—and 16, 32, and 64 channels (feature maps), respectively. Stride and padding were assigned a value of 1. ReLu and max-pooling layers were inserted between convolutional layers. The classification output was preceded by a SoftMax and fully connected (FC) layer. Convolutional and FC layers had trainable parameters. These parameters were used also in simulations with the 2-conv shallow network. For simulations with movement signals added as a global scalar before the second or third convolutional layers, we inserted an “addition layer” with a feature input tensor of a size commensurate with the convolutional layer. For simulations with modulations in movement patterns overlapping with visual inputs, we also added a feature tensor right after the input layer but set all input values to zero. In all four main architectures, we added a trainable batch-normalization layer after the convolution layer that received movement inputs. AlexNet was downloaded as a Matlab implementation (Deep Neural Networks Toolbox).

### Input stimuli

Input stimuli in simulations with fixational eye movements were grayscale images (28×28 px, [0,1] amplitude range) representing simplified V1 retinotopic activity patterns elicited by horizontal luminance bars with changing elevations (7 px H, 20px W, no overlap). Simplified movement-related activations were simulated as rectangular activity patterns (21 px H, 20 px W). We used one movement pattern for simulations with eye movements only and two patterns (14 px overlap) for simulations of S2 Fig. In the latter simulations, we used four stimuli corrupted by random pixel noise and their amplitude was modulated to mimic 10 contrast levels ([0,1] with 0.1 step size) to help minimize overfitting during training. In the simulations with eye movements, we used three vertically separated visual stimuli. Movement patterns had a significant spatial overlap with visual stimuli (12 px) and differed from each other (and from the visual stimuli) as also observed in neural data [70].

### Eye movements with luminance bars

We simulated eye movements by shifting upward or downward the (cortical) activations associated with the three luminance bars in visual space, thereby allowing for five possible ocular shifts. We divided the input image into five (equally high and nonoverlapping) horizontal bands, with *default* locations (for no eye movements) in bands 1, 3, and 5. When the central stimulus was displayed in the visual space and the eyes had not moved, the evoked response was in band 3. In the absence of eye movements, bands 1 and 5 were activated by the top and bottom stimuli in visual space, respectively. Instead, if the eyes had moved, depending on the movement size (randomized), a stimulus in visual space could activate any of the five bands. For example, the central bar (vertical band 3) could shift by [0, ±1, ±2] locations; a shift of +1 would correspond to band 2, whereas a shift of +2 would correspond to the top band 1, that is, the same band of the top stimulus with no eye movements (Fig 1C). The top and bottom stimuli could only be shifted downward or upward, respectively, with a location degeneracy relative to the other stimuli. For simulations in which the amplitude of the motor activations could change, the amplitude varied between five levels from a reference value of 0.5, with increments and decrements (including zero amplitude) depending on movement size and direction: downward movements, negative amplitude decrements and upward movements positive increments. Amplitude changes in [-0.8, +0.8] range, with a step size of 0.2. Therefore, composite images with visual and movement stimuli were in the [-1.3, +1.3] amplitude range. In pixels where visual and motor inputs overlapped, summation was sub-linear, as found experimentally [70]—here implemented as a clipping to the max value between the two signals for simplicity.

### Optimization

The optimization of trainable parameters was based on categorical cross-entropy loss, regularized with early-stopping to avoid overfitting. Optimization was stopped at approximately 80% accurate classification for fixational eye movement simulation (chance level 33%) and approximately at 20% for saccadic eye movement simulations (chance level 10%). Optimization parameters (e.g., learning rate and batch size) were set to be equal for all simulations to enable interpretable relative comparisons. All results focused on *relative* accuracy changes, which were not qualitatively affected by the asymptotic performance of the trained network. Training was run on a GPU server with 8 Nvidia RTX2080-Ti GPUs (ASRok motherboard 3U8G+/C621) using MATLAB Parallel Computing Toolbox with Slurm plugin.

### Network training and testing

For fixational eye movement simulations, training and testing sets had 800 samples with a 50% partition. Reported error bars are standard deviations across test sets, except in Fig 1F, in which the variability is estimated across 20 random initializations of the network. For simulations with the modified CIFAR-10 database, we used 50,000 training examples and 10,000 validation examples. Early stopping was implemented by limiting the training epochs to 15; the number of epochs was set to 55 when allowing congruent and incongruent simulations to reach plateau performance. We typically ran 10 training interactions of the same network to account for the variability caused by the randomization of the initial weights. The mini-batch size was set to 64 and the validation frequency to 700. Optimization typically terminated with matching training and validation performance, thereby suggesting no significant overfitting. Performance was always evaluated on held-out trials.

### Electrical stimulation

Simulations of focal external stimulations (electrical stimulation, S3 Fig) were done using a network trained with two movement patterns and four visual stimuli, while testing it with a small activity pattern (3×3 px) with high contrast values (level 10, see “Input stimuli”). The results were qualitatively stable relative to small changes in activity locations, sizes, and contrast levels of the luminance spot.

### Modified CIFAR-10

The CIFAR-10 database was downloaded from https://www.cs.toronto.edu/~kriz/cifar.html. Each image was first converted to BW, the edges (3 px wide) were replaced by random noise ([0, 255] amplitude range) and a frame or random noise was added around each side of the image (5 px wide). Image shifts were implemented as circular x-y shifts of random amplitudes in the range [-8, +8] px. In simulations with the shallow 2-conv layer network smaller saccadic shifts were in the range [-4, +4], and [-2, +2].

### Receptive field analysis

We analyzed networks trained with modified BW CIFAR-10 images, but with no saccadic shifts (shift range = [0, 0]). We then used the MATLAB function deepDreamImage.m (Deep Learning Toolbox, R2021a) with 2 iteration levels and 2 pyramid levels. Because this function does not accept networks with multiple inputs, after training we disassembled and reassembled the network and excluded the movement-input layer; this procedure preserved learned weights across all other layers. For the plots in S5 Fig, we handpicked the representative examples.

### Hyper-orientation–discrimination acuity

Oriented sinusoidal grating had the same size and amplitude range as the modified CIFAR-10 images, with six different angles in the [0, pi] range. Example gratings with the same orientation differed from each other due to the randomization of the grating spatial phase and the random noise in the surrounding frame (see methods to modify CIFAR-10 images). Networks (n = 10) were trained with a very small proportion of grating stimuli, with as little as 0.05% resulting in just a handful of examples for each orientation. With higher proportions, networks would quickly achieve near-optimal classification accuracy. The network output was modified in view of the larger classification set (16 output classes). We then tested these trained networks with new datastores constructed primarily of grating stimuli (90%). For the PCA analysis, in each layer, we computed activations for each channel and averaged across pixel space (receptive field) using a balanced data set relative to the representative angles. On the first three PCs, we fitted a linear discriminant model (fitcdiscr.m Matlab function) with five-fold cross validation. For the tuning curves, we computed mean and 5%-95% confidence interval across examples of gratings with the same orientation. Responses were computed as pixel-average across the entire receptive field. Proportional changes in responsive neurons across layers were computed as the proportion of channels whose maximum amplitude in the tuning curve exceeded an arbitrary threshold of 0.1.

### Congruent and incongruent movement inputs

Movement inputs were added as a “feature” layer, immediately before a target convolutional layer (conv-layer 1, and in controls for layer dependence of classification accuracy also conv-layer 2 and 3). Their matrix/tensorial dimensions varied to match the dimension of each target layer. In most simulations, they were added before conv-1 and had the same dimensions as the input images; 20% of pixel values were set to zero, while the remaining pixels were assigned either saccadic-vector values $\left(S_{1}^{i},\;S_{2}^{i},\right)$ used to saccadic-shift image *I_i_* or a pair of random values within the saccadic range. This was done via a 40%-40% pixel split, with random assignment relative to pixel indices. Movement images were first generated with amplitude values in an interval [-8, +8] px—that is, the range of saccadic shifts. Then, depending on the layer where they were added, amplitudes were rescaled to match those of visual inputs. For example, when added to the first convolutional layer, movement amplitudes were rescaled to be 25% of the image amplitudes in conv-1. To determine the range of amplitudes of CIFAR-10 images at various layers, we initialized the network using only one training epoch and computed minimum and maximum amplitudes at each layer. In simulations with the main CNN architecture but smaller movement amplitudes, the downscaling was 10%, and 5% for smaller amplitudes in shallow-network simulations. Saccadic amplitudes were allowed to assume negative values, while visual inputs only positive ones. Pixel values in movement images reflected the (x,y) scalar values used to shift the CIFAR-10 images, but did not carry information on which scalar corresponded to the x- or y-shift. Missing this information, the network could only access “quadrants” information (as in Cartesian quadrants): for example, two positive scalars indicate shifts in the 1st quadrant, while two negative scalars indicate shifts in the 3rd quadrant. Scalars with different sign indicate shifts in either the 2nd or 4th quadrant, which overall provides above chance-level information for quadrant assignments, with 75% maximum achievable performance. In Fig 2E, an SVM classifier was used to separate 3 conditions: 1^st^ quadrant, 3^rd^ quadrant, and 2^nd^ or 4^th^ quadrant, hence with 100% maximum achievable performance.

### Decoding saccadic directions

As explained above, movement inputs were provided to the network as indexed images whose values reflected saccadic-shift coordinates. Therefore, from movement images it was possible to extract shift values, but not the information of which of the two scalars represented the horizontal and vertical saccadic shift. Therefore, movement images could be used to define four saccadic-direction groups: (i) cardinal shifts along the x or y axis, i.e. (0, s) or (s, 0), with ‘s’ a non-zero, signed scalar; (ii) 1^st^ quadrant shifts, i.e. (s1, s2), with s1 and s2 both positive scalars; (iii) 3^rd^ quadrant shifts, i.e. (s1, s2), with s1 and s2 both negative scalars; (iv) 2^nd^ and 4^th^ quadrants shifts, i.e. (s1, s2), with s1 and s2 scalars with different sign; To examine the network’s classification accuracy for direction groups, we considered channels’ activations to movement inputs only, we reduced their dimensionality using PCA (n = 3), and used a 5-fold, cross-validated SVM linear classifier. We did so at each layer and separately for networks that did and did not exceed chance-level performance in image classification. Significance values reported are p-value of a two-sided Wilcoxon rank sum test.

### Activity modes and angular separation between stimulus-movement patterns

We tested networks trained with congruent movement input using either visual stimuli or movement stimuli. We did so using n = 1000 example images separately in networks that did and did not exceed chance-level performance. For each example image, network, and layer, we obtained two vectors of activations across channels (A,B), one vector for movement inputs and one for visual inputs, and computed the angle between them as acos(abs(A’*B)). For each network and layer, we then averaged angles across examples. Statistical testing (unpaired t-test) was conducted between networks (at or above chance level performance) on example-averaged angles.

### t-SNE and SVM classifier

We considered networks trained with congruent movement conditions and computed channel activations at each layer using a datastore with only visual inputs, zeroing movement inputs. For each channel, we computed a mean activation value to each sample image as the mean across channel RF pixels (units). On this [channels x image samples] response matrix, we applied t-SNE (Euclidean distance, perplexity = 30). Finally, on this reduced dimensionality data set, we trained a quadratic SVM model using the CIFAR-10 categorical labels and five-fold cross-validation. In simulations with networks trained with congruent movements and tested with saccadic image shifts and either congruent or incongruent movements, instead of t-SNE we used PCA to reduce the dimensionality to 20 components.

### Saccadic suppression, enhancement, and gain fields

The test stimulus was an image with a luminance square in the center, 5 px in size. The luminance value was set to be the median amplitude value across all images in the training set. We then grouped movement inputs from the validation set based on the vector length for the corresponding saccadic shift: $\sqrt{S_{1}^{2}+S_{2}^{2}}$ and assigned the movement input to one of six saccadic-amplitude groups, linearly spaced from zero to max vector length; each group was populated with the same number of example movements (n = 20). Movement + visual input datastores were then used to measure neuron activations in each layer. A single activation value was computed for each channel by averaging across pixels in the portion of the receptive field covering the luminance square, which changed in size from layer to layer. The response change was computed as the difference between the average response with movement + visual input and the response with only the visual input. In Fig 4 variability was computed as the standard deviation across channels of the response change, thus with a variability value associated with each saccadic input. In the summary plot of Fig 4D, for each network and saccadic interval, we computed variability values as the amplitude of the 5^th^–95^th^ confidence interval for the associated s.d. values. Thereafter, we made a scatter plot of these values for networks at and above chance-level performance and repeated this procedure for each layer.

## Supporting information

S1 Table Basic CNN architecture and parameters. This template architecture was modified for different simulations with movement-related inputs added before either the conv-2 or conv-3 layers. Similarly, only one “batchnorm” layer was utilized in simulations with movement inputs in deep layers, as detailed in S1 Fig.
(DOCX)

S1 Fig Network architectures used in simulations. The six architectures used in simulations with titles referring to the corresponding sections in the main text. Details of the parameters used for each layer are in S1 Table. The features input is the movement-related input implemented as a tensor of size commensurate with the following convolutional layer. The ‘add_1’ layer is an addition layer (additionLayer function, Deep Learning Toolbox, Matlab R2020b).
(EPS)

S2 Fig Generation of movement images. **a**) Example of a modified CIFAR-10 image, BW, with a noisy frame and a bottom-left shift (red arrows and vector in the 3rd quadrant) according to (x,y) = (-7,+3) shift scalars in red. **b**) Shift values are multiplied by the scaling factor “SCL” and randomly assigned to an equally sized image having 20% of its pixels set to zero, 40% set to the scaled x-shift value, and 40% to the scaled y-shift value. The movement image does not retain information about which scalar was used for the x or y shift, only that the shift vector was in the 3rd quadrant (2nd and 4th quadrant vectors are therefore indistinguishable from the pixel values in the movement image).
(TIF)

S3 Fig Classification invariance and phosphenes. **a**) Example of visual and movement-evoked activations used to train the CNN (s, stimuli; m, movements). Random pixel noise and amplitude changes simulate neural variability, changes in visual contrast, and amplitude modulations linked to different movement sizes (introduced to reduce overfitting; Methods). **b**) The CNN can easily learn to distinctively classify visual and motor inputs (error bars, s.d. across test stimuli, with early stopping during training at approximately 80% performance; Methods). **c**) Testing the trained network with a small luminance spot (dissimilar from all trained patterns) at various image (cortical) locations produces output probabilities across all channels reflecting overall classification uncertainty and a significant probability of reporting a visual input (integral across all visual channels).
(EPS)

S4 Fig Modified CIFAR-10 database. **a**) Original CIFAR-10 database, 10 randomly selected images. **b**) Modified CIFAR-10 database used in this study. Briefly, images were converted to BW, framed with random noise, and shifted to mimic saccadic eye movements. Titles show the x and y shifts in the range [-8, +8] px. Images were not “circularly shifted” to void breaking the overall spatial coherence of the represented elements.
(EPS)

S5 Fig Discrimination of oriented stimuli. **a**) Example of CIFAR-10 images with a random-noise frame and with no saccadic shift (compare with S3 Fig). **b**) Representative examples of receptive fields (DeepDream activations, Methods) for cells at different layers. **c**) An example of a grating stimulus used to train and test the network. As implemented with CIFAR-10 images, framing noise changed across examples of the same orientation as well as the spatial phase of the grating. **d**) Performance of CNNs (n = 30) trained with a large proportion of CIFAR-10 example images and a small proportion of grating stimuli (filled blue bars, error bars, s.e.). Empty bar represents the performance of the LDA classifier on PCA representations; dotted horizontal line represents the chance-level performance. **e**) Top three PCs of population responses to oriented gratings in different layers. Color code for the six presented orientations. LDA performance for these clustered representations is optimal at all layers (as shown in panel **d**, empty bar). Good decoding also in L1 suggests that even subtle amplitude differences in largely circular RFs are sufficient to achieve optimal decoding accuracy, as shown in the next panel. **f**) Example orientation tuning curves (columns) for cells in different CNN layers (rows); note the smaller y-scale for two example cells in layer 3. **g)** Proportion of cells with a significant orientation tuning in each layer. The proportion collapses in L3, which is in agreement with the poor overall classification performance of the network for grating stimuli (panel **d**).
(EPS)

S6 Fig Confusion matrices at different performance levels. Confusion matrices detail the classification performance of a trained network showing true vs predicted classes. Red vertical “stripes” indicate the network is overclassifying a specific class (bottom example, airplane). A uniformly red-ish matrix (top example) indicates a network that on average correctly classifies the various classes but with a fair degree of variability. Marginals for diagonal and off-diagonal classifications are color coded at the bottom and right side of each matrix. As the performance degrades, the tendency for single-class overclassification increases.
(EPS)

S7 Fig Confusion matrices of SVM classifiers. Left column, confusion matrices for a quadratic SVM classifier trained on t-SNE representations across layers (rows, and titles). This example network reached an overall accuracy of 25%. Right column, similar confusion matrices for a linear SVM trained on PCA representations. In this representative example, PCA had a tendency to over-classifying a reduced set of classes across all layers, which was less obvious in t-SNE representations, especially for L3 (bottom-left matrix).
(EPS)
