# Supplementary material for: Motor-related signals support localization invariance for stable visual perception
Source: PLoS Comput Biol. 2022 Mar 14;18(3):e1009928. doi: 10.1371/journal.pcbi.1009928 (PMC8947590; doi:10.1371/journal.pcbi.1009928)
Supplement: S1 Table — This template architecture was modified for different simulations with movement-related inputs added before either the conv-2 or conv-3 layers. Similarly, only one “batchnorm” layer was utilized in simulations with movement inputs in deep layers, as detailed in S1 Fig. (DOCX) [file pcbi.1009928.s001.docx]

| 1 | “image input” | Image Input | 28x28x1 images with “zero-center” normalization |
| --- | --- | --- | --- |
| 2 | “conv_1” | Convolution | 16 3x3x1 convolutions with stride [1 1] and padding [1 1 1 1] |
| 3 | 'batchnorm_1' | Batch Normalization | Batch normalization with 16 channels |
| 4 | “relu_1” | ReLU | ReLU |
| 5 | “maxpool_1” | Max Pooling | 2x2 max pooling with stride [2 2] and padding [0 0 0 0] |
| 6 | “conv_2” | Convolution | 32 3x3x16 convolutions with stride [1 1] and padding [1 1 1 1] |
| 7 | “batchnorm_2” | Batch Normalization | Batch normalization with 32 channels |
| 8 | “relu_2” | ReLU | ReLU |
| 9 | “maxpool_2” | Max Pooling | 2x2 max pooling with stride [2 2] and padding [0 0 0 0] |
| 10 | “conv_3” | Convolution | 64 3x3x32 convolutions with stride [1 1] and padding [1 1 1 1] |
| 11 | “batchnorm_3” | Batch Normalization | Batch normalization with 64 channels |
| 12 | “relu_3” | ReLU | ReLU |
| 13 | “fc” | Fully Connected | 6 fully connected layer |
| 14 | “softmax” | Softmax | softmax |
| 15 | “class output” | Classification Output | with “1” and 5 other classes, Loss Fcn: cross entropy |
