# Supplementary figures and images for: Motor-related signals support localization invariance for stable visual perception
Source: PLoS Comput Biol. 2022 Mar 14;18(3):e1009928. doi: 10.1371/journal.pcbi.1009928 (PMC8947590; doi:10.1371/journal.pcbi.1009928)

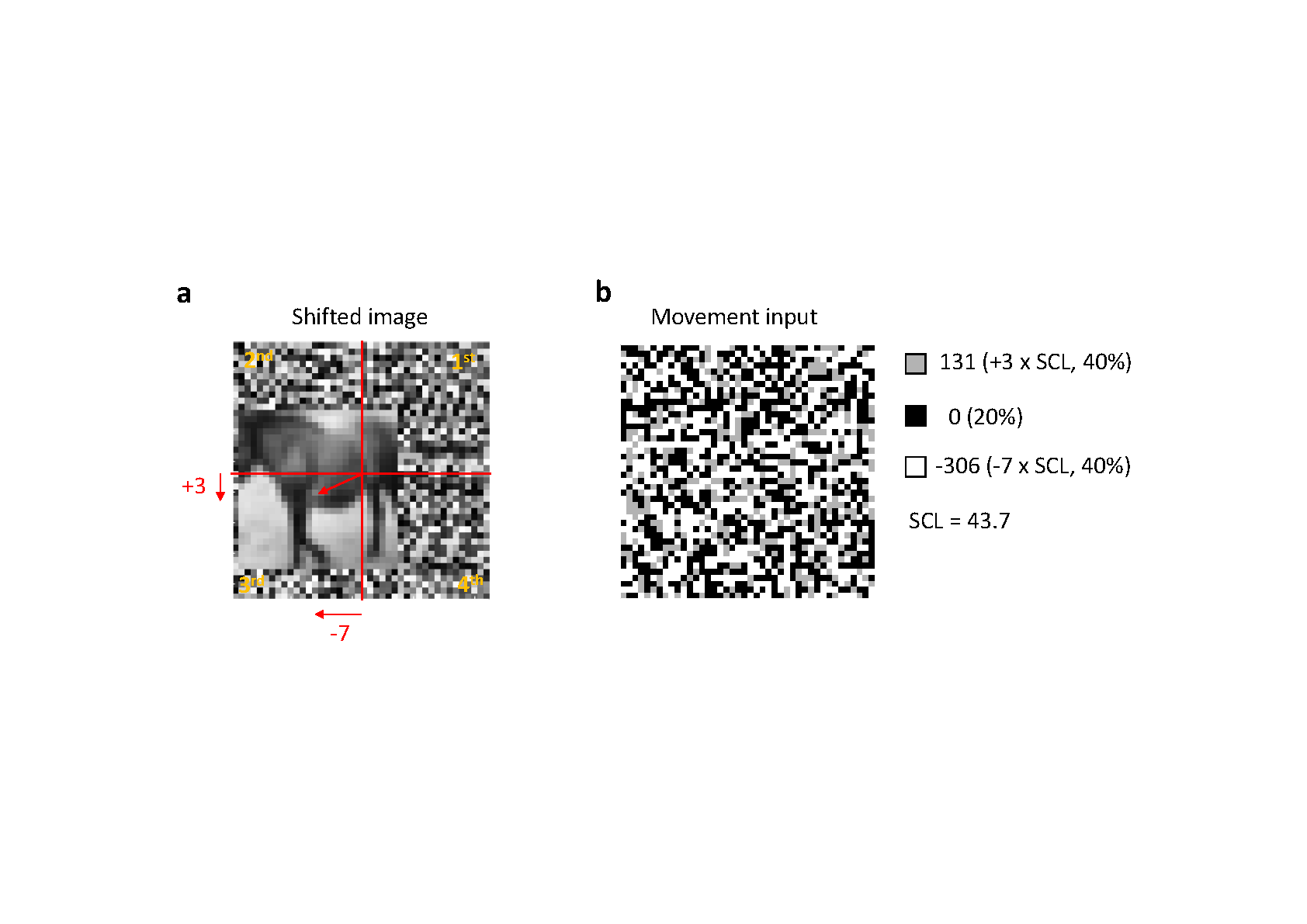

Supplement: S2 Fig — a) Example of a modified CIFAR-10 image, BW, with a noisy frame and a bottom-left shift (red arrows and vector in the 3rd quadrant) according to (x,y) = (-7,+3) shift scalars in red. b) Shift values are multiplied by the scaling factor “SCL” and randomly assigned to an equally sized image having 20% of its pixels set to zero, 40% set to the scaled x-shift value, and 40% to the scaled y-shift value. The movement image does not retain information about which scalar was used for the x or y shift, only that the shift vector was in the 3rd quadrant (2nd and 4th quadrant vectors are therefore indistinguishable from the pixel values in the movement image). (TIF) [file pcbi.1009928.s003.tif]
